# Alkylresorcinols activate SIRT1 and delay ageing in *Drosophila melanogaster*

**DOI:** 10.1038/srep43679

**Published:** 2017-03-02

**Authors:** Yasunari Kayashima, Yuki Katayanagi, Keiko Tanaka, Ryuta Fukutomi, Shigeru Hiramoto, Shinjiro Imai

**Affiliations:** 1 Department of Food and Nutrition, Yamanashi Gakuin Junior College, 2-4-5 Sakaori, Kofu-shi, Yamanashi 400-8575, Japan; 2 Health Care Research Center, Nisshin Pharma Inc., 5-3-1, Fujimino, Saitama 356-8511, Japan; 3 School of Bioscience and Biotechnology, Tokyo University of Technology, 1404-1, Katakura, Hachioji Tokyo 192-0982, Japan.

## Abstract

Sirtuins are enzymes that catalyze NAD+ dependent protein deacetylation. The natural polyphenolic compound resveratrol received renewed interest when recent findings implicated resveratrol as a potent SIRT1 activator capable of mimicking the effects of calorie restriction. However, resveratrol directly interacts with fluorophore-containing peptide substrates. It was demonstrated that the SIRT1 activation of resveratrol is affected by the amino acid composition of the substrate. Resveratrol did increase the enzyme activity in cases in which hydrophobic amino acids are at the +1 position to the acetylated lysine in the substrate. Alkylresorcinols (ARs) are compounds that belong to the family of phenolic lipids, and they are found in numerous biological species. Here we show that the natural activators ARs increased the V_max_ of recombinant SIRT1 for NAD+ and peptide substrate, and that ARs decreased acetylated histone in human monocyte cells by stimulating SIRT1-dependent deacetylation of substrates. ARs also extended the lifespan of *Drosophila melanogaster*, which was shown to be dependent on functional *Sir2*. Our results demonstrated that ARs are natural catalytic activators for sirtuin.

Sir2-like proteins (sirtuins) are a family of NAD+ -dependent protein deacetylases conserved from *Escherichia coli* to humans^1^. Sirtuins play important roles in gene silencing, DNA repair^2^, rDNA recombination^3^, and ageing in model organisms^4^,^5^,^6^. In diverse species, the lifespan is extended when calories in the diet are restricted, suggesting that there is a conserved mechanism for the nutrient regulation of aging^7^,^8^,^9^. Several classes of polyphenols — including chalcones, flavones, and stilbenes — increase the rate of deacetylation for acetylated peptide substrate. The polyphenol compound resveratrol has been shown to stimulate deacetylation^10^,^11^ in a dose-dependent manner up to 2.5-fold for acetylated SIR-2.1 peptide and 2.4-fold for acetylated *Sir2* peptide. Increased Sirt1 activity, provided by either a transgenic overexpression of *Sirt1* gene in mice^12^ or through pharmacological activation by resveratrol, has been shown to have beneficial effects on type 2 diabetes in rodent models^13^, indicating that the protein SIRT1 (sirtuin 1) may represent an attractive therapeutic target.

However, SIRT1 activation by resveratrol with a coumarin-labeled peptide is the result of a resveratrol-induced conformational change near the coumarin binding site in SIRT1, which creates a binding pocket for the coumarin group, resulting in enhanced binding of the coumarin-labeled peptide^14^. SIRT1 activation with resveratrol has been assessed by biochemical assays utilising native substrates, including a p53-derived peptide substrate lacking a fluorophore, as well as the purified native full-length protein substrate p53. The use of resveratrol does not lead to an apparent activation of SIRT1 with native peptide or full-length protein substrates, whereas it does activate SIRT1 with a peptide substrate containing a covalently attached fluorophore^15^. Resveratrol activates the enzyme activity in cases in which the aromatic amino acids are at the +1 position of the acetylated lysine in the substrate^16^. Regardless of the primary structure of the sirtuin substrate, natural catalytic activators for sirtuin have not yet been identified.

Sirtuin has physiologic functions that are exerted regardless of the differences between males and females, but resveratrol was shown to extend the lifespan of male but not female *Drosophila melanogaster*^11^. An activator of sirtuin could thus be expected to extend the lifespan of these flies regardless of the sex differences. We therefore screened a number of phytochemical libraries for a SIRT1 activator. A deacetylation activity measurement kit that does not give pseudo-positive fluorescence results was developed by CycLex^17^ (Ngano). In our experiment, resveratrol does not have an intrinsic deacetylation activity by this kit_ (data not shown).

In the present study, we screened a number of phytochemical libraries for a SIRT1 activator using the CycLex kit, and we identified resorcinolic lipids as possible candidate substance. Resorcinolic lipids are non-isoprenoic phenolic lipids with analogues ranging from C5–C25, and they are found at high concentrations in whole grain wheat and rye^18^. Alkylresorcinols (ARs) have received a great deal of attention from food and nutrition researchers because of the suggested health benefits^19^. ARs are found in grain husk such as that of wheat or rye at concentrations between 300 and 1500 μg/g^19^. ARs have been observed to have bioactivities in many *in vitro* models, and grain husk ingestion may be important in food and human nutrition^19^. ARs are absorbed from the human intestine, and their plasma concentrations are maintained as short-to-medium-term biomarkers of the intake of whole-grain wheat and rye, because the estimated half-life of plasma ARs is <5 hr^20^.

Rats fed ARs at 4 g/kg diet had elevated γ-tocopherol and reduced total cholesterol concentrations in the liver^21^. ARs increased glucose tolerance by suppressing hepatic lipid accumulation and intestinal cholesterol absorption, which subsequently suppressed diet-induced obesity in mice^22^. Because reseveratrol also increases glucose tolerance in this manner^23^, this function may be related to the activation of sirtuins by ARs.

## Results

**Figure 1:**
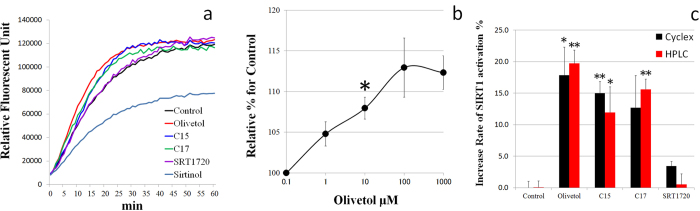
The effects of the ARs on recombinant SIRT1. (**a**) The time course of SIRT1 deacetylation activity of ARs. ARs and SRT1720 were assessed at 10 μM, and sirtinol was assessed at 100 μM by recombinant SIRT1 with 25 μM of NAD+. All values are the mean of three determinations. (**b**) The dose-dependency of olivetol for SIRT1 activity with 100 μM of NAD+ (±standard error [s.e.]) at 1000 sec. All values are the mean of at least four determinations. (**c**) The rate of the increase of SIRT1 activation with native p53 peptide monitored by the HPLC assay and CycLex assay at 1000 sec. All values are the mean of three determinations. Errors = s.e. *p < 0.05, **p < 0.01, compared with the control.

The results of the CycLex assay confirmed that (*1*) the ARs activated the deacetylation activity of SIRT1, (*2*) the small-molecule SIRT1 activator SRT1720^13^ did not activate SIRT1, and (*3*) 100 μM of sirtinol inhibited the deacetylation activity of SIRT1 (Fig. 1a). Because long-alkyl ARs are indissoluble in water, we could not determine the concentration-dependency of the SIRT1 activation by the long-alkyl ARs. There are many types of ARs. Olivetol (5-pentylresorcinol), which is a short-alkyl chain AR, is slightly soluble in water. We investigated whether olivetol can activate SIRT1. As shown in Fig. 1b, olivetol showed dose-dependent SIRT1 deacetylation activity between 1 μM and 100 μM. We performed a non-fluorescent deacetylation assay using a separation of the acetylated peptide substrate and the deacetylated peptide product by reverse-phase HPLC. The ARs series and SRT1720 were assessed at 10 μM. The results demonstrated that the ARs activated SIRT1 at the same levels as those shown by the CycLex assay results except for SRT1720 (Fig. 1c).

**Figure 2:**
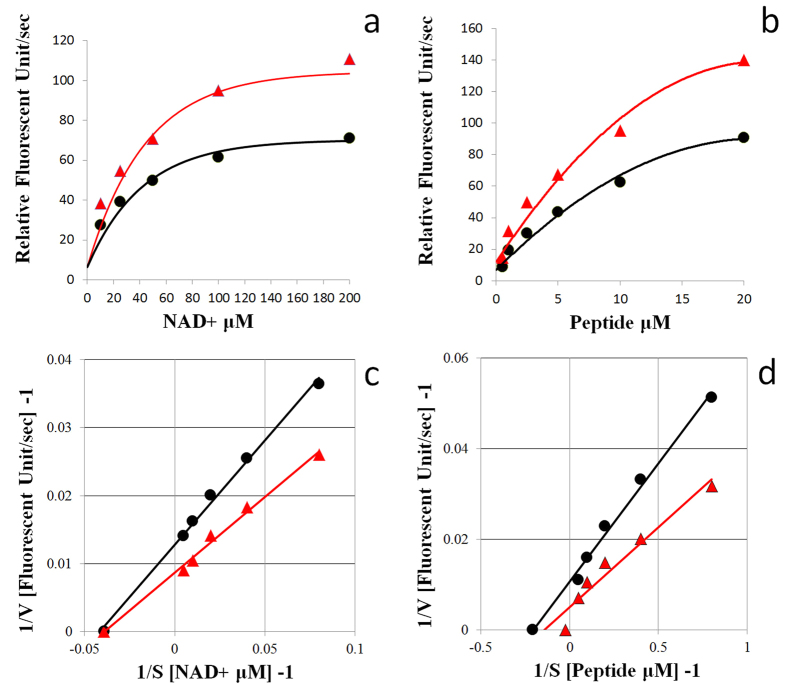
Michaelis-Menten plot of C17:0-AR for SIRT1 activity with various concentrations (12.5–200 μM) of NAD+ at 1000 sec (**a**) and with various concentrations (0.5–20 μM) of peptide substrate at 1000 sec (**b**). Lineweaver-Burk plot of C17:0-AR for SIRT1 activity with various concentrations (12.5–200 μM) of NAD+ at 1000 sec (**c**) and with various concentrations (0.5–20 μM) of peptide substrate at 1000 sec (**d**). Black lines: control. Red lines: C17:0-AR. All values are the mean of at least three determinations.

We plotted the results of our kinetic studies of SIRT1 activation for the control and C17:0-AR (10 μM) as a Michaelis-Menten graph. C17:0-AR had no significant effect on the determination of the Michaelis constants (K_m_ = V_max_/2; Control: 24.0 μM; C17:0-AR: 25.6 μM) when NAD+ was varied, but it had pronounced effects on the apparent V_max_ (Control: 78.1 unit/sec; C17:0-AR: 114.9 unit/sec). C17:0-AR raised the V_max_ for NAD+ by 1.47-fold (Fig. 2a). C17:0-AR had no significant effect on the Michaelis constants (Control: 6.9 μM; C17:0-AR: 4.9 μM) when the peptide substrate was varied, but it had pronounced effects on the apparent V_max_ (Control: 93.5 unit/sec; C17:0-AR: 196.1 unit/sec). C17:0-AR raised the V_max_ for peptide substrate by 2.10-fold (Fig. 2b). The Lineweaver-Burk plot indicated that while the x-intercept was the same, the slopes and the V_max_ differed between the control and C17:0-AR (Fig. 1c,d).

**Figure 3:**
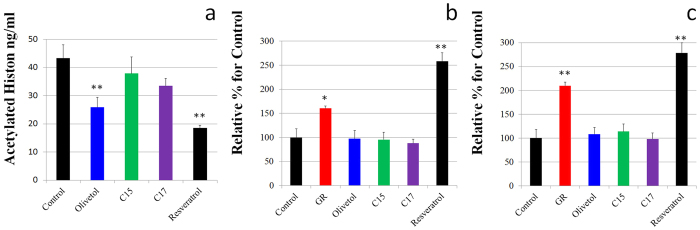
Effects of ARs on human monocyte cells. (**a**) Effect of ARs (10 μM) on TSA-insensitive deacetylase activity for histone in THP-1 cells. (**b**) Effect of ARs (10 μM) and GR condition on the mRNA expression levels of SIRT1 in THP-1 cells. (**c**) Effect of ARs (10 μM) and GR condition on the mRNA expression levels of SIRT1 in HepG2 cells. Errors = s.e. *p < 0.05, **p < 0.01 compared with the control. All values are the mean of at least three determinations.

To establish whether ARs can activate sirtuins in mammal cells, we performed a cell-based deacetylation assay using human cell lines. Unlike other classes of deacetylases, the sirtuins are insensitive to the inhibitor trichostatin A (TSA). ARs, including olivetol and resveratrol, decreased the rate of TSA-insensitive acetylated histone (Fig. 3a). Although resveratrol does not directly activate deacetylation activity of SIRT1, it extends the lifespan of animals without reducing fecundity. Resveratrol also significantly modulated the SIRT1 mRNA pattern in mammal cells^24^.

To establish whether ARs can modulate the SIRT1 mRNA in mammal cells, we examined the expression levels of SIRT1 mRNA in AR-stimulated cell lines. Resveratrol and glucose restriction (GR) culture condition as an alternative caloric restriction^25^ significantly up-regulated the expression levels of SIRT1 mRNA in the human monocyte cell line THP-1 and the human liver cancer cell line HepG2. However, the ARs did not affect the gene expression levels of SIRT1 mRNA (Fig. 3b).

**Figure 4:**
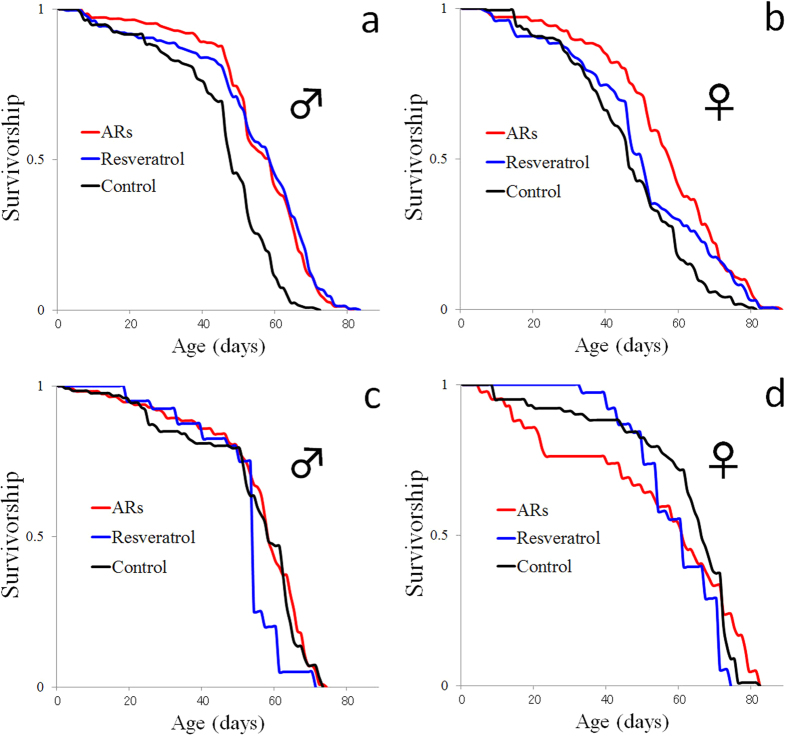
Survival of wild-type and Sir2-deficient genotype *D. melanogaster* adults fed an ARs or resveratrol. Males (**a**) and females (**b**) of the wild-type *w*^*1118*^ line. Males (**c**) and females (**d**) with the strong hypomorphic genotype *Sir2*^*17*^/*Sir2*^*KG00871*^.

We investigated whether ARs can extend the lifespan of *D. melanogaster*. Shortly after they reached adulthood, wild-type flies (*w*^*1118*^ line) were transferred to culture vials containing 0.07% (18.6 mM equivalent) of an AR mixture (1,3-dihydroxy-5-heptadecylbenzene [C17:0], 33.1%; 1,3-dihydroxy-5-nonadecylbenzene [C19:0] 33.6%; 1,3-dihydroxy-5-heneicosylbenzene [C21:0], 25.3%; and 1,3-dihydroxy-5-tricosylbenzene [C23:0], 8.0%) or 100 μM of resveratrol in SF medium, under the demographic culturing conditions described in the Methods section. Across the independent tests in males and females on an abundant diet, the lifespan was extended up to 22.0% with ARs and up to 19.0% with resveratrol (Fig. 4a) in male, the lifespans of the females were extended by 21.8% with ARs and up to 7.7% with resveratrol (Fig. 4b). The median lifespan of the three male groups was 44.6 days (SF), 54.4 days (ARs) and 53.1 days (resveratrol). The median lifespan of the three female groups was 45.2 days (SF), 55.0 days (ARs) and 48.6 days (resveratrol) (Table 1). These data suggest that ARs and resveratrol extend the lifespan of *D. melanogaster*. There were no significant differences in food uptake as analysed by a capillary feeder (CAFE) assay^26^ between the AR-fed flies and the SF-fed flies (data not shown).

To determine whether the ARs could extend the flies’ lifespan in a Sir2-dependent manner, we analysed a *Sir2* allelic series with increasing amounts of *Sir2*. Adult offspring from crosses between independently derived alleles of *Sir2* were tested. The ARs and resveratrol failed to extend the lifespan in the flies in which *Sir2* was severely decreased (*Sir2*^*17*^/*Sir2*^*KG00871*^) (Fig. 4c,d, Table 1). These data suggest that the ability of ARs to extend the *D. melanogaster* lifespan requires functional *Sir2*.

## Discussion

Our experiments demonstrated that human SIRT1 deacetylase activities were increased with ARs. In the CycLex assay, 100 μM of sirtinol inhibited the deacetylation activity of SIRT1, and the inhibition rate was approx. 40%, which corresponds to the reported^27^ IC_50_ value (131 ± 11 *μ*M) of sirtinol. This result indicates that the CycLex assay accurately measures the deacetylation activity of SIRT1. The results of our kinetic studies indicate that ARs increase the catalytic activity of SIRT1 by affecting the enzyme structure. This effect may be an allosteric SIRT1 activation by ARs.

We observed that the ARs and resveratrol decreased the rate of TSA-insensitive acetylated histone in the human monocyte cell line THP-1. Unlike other classes of deacetylases, sirtuins are insensitive to the inhibitor TSA. Olivetol, C17:0-AR, and resveratrol significantly decreased the rate of acetylated histone, indicating that ARs are able to activate sirtuins in mammalian cells. In 2010, resveratrol was reported to directly interact with fluorophore-containing peptide substrates^15^. After that report, the direct mode of SIRT1 activation by resveratrol gained renewed support. The fluorescent moiety on substrates is indeed dispensable for activation if it is replaced by hydrophobic amino acids^28^. Native peptide substrates such as PGC-1α were shown to mediate activation by resveratrol *in vitro*^16^, and PGC-1α contain alanine residues at the +1 position relative to the acetylated lysine, the same positions as the fluorophores in the original assays^10^,^13^. These results demonstrated that there are structural and positional requirements in natural substrates that mediate SIRT1 activation.

The substrate that we used has a leucine residue at the +1 position relative to the acetylated lysine. The ARs showed activation of SIRT1 at the same levels as those revealed by the CycLex assay, but this was not true of resveratrol. In our experiments, SRT1720 did not induce the deacetylation activity of SIRT1 in the CycLex or HPLC assays. SRT1720 activity may affect SIRT1 activation based on the amino acid composition of the substrate.

Although resveratrol did not directly induce the deacetylation activity of SIRT1, resveratrol has been reported to extend the lifespan of animals without reducing fecundity, and it significantly modulated the SIRT1 mRNA pattern in mammalian cells^24^,^29^. Although caloric restriction has been shown to increase lifespan in various animal models, the mechanisms underlying this phenomenon have not yet been revealed. *In vitro* system to mimic caloric restriction by reducing glucose concentration in cell growth medium was developed^25^. In this system, GR in human fetal lung fibroblasts cells up-regulated gene expression levels of the SIRT1. We examined whether GR in THP-1 and HepG2 cells affect gene expression levels of the SIRT1. GR in both cells increased gene expression levels of the SIRT1 compared with normal glucose concentration control. In the present study, resveratrol significantly up-regulated the expression levels of SIRT1 mRNA in the human monocyte cell line THP-1 and the human liver cancer cell line HepG2, whereas the ARs did not affect the expression levels of SIRT1 mRNA. These data suggest that ARs up-regulate the histone deacetylation without modulating the SIRT1 mRNA expression.

ARs increase the lifespan in *D. melanogaster* in a Sir2-dependent manner, and this action appears to function through the other pathway related to caloric restriction. The overexpression of sirtuins has been reported to increase the lifespan in *D. melanogaster*^30^. However, when the reported effects of sirtuin overexpression on ageing were closely re-examined, it was found that standardization of the genetic background and the use of appropriate controls abolished the apparent effects in *D. melanogaster*^31^, because longevity depends on sex and genetic background^32^. In contrast, when an inducible gene switch system was used for the conditional expression of *Sir2*, the *D. melanogaster* lifespan was increased^33^. Because sirtuin activation levels with ARs are mild, the lifespan of *D. melanogaster* might be extended.

Resveratrol does not directly activate SIRT1 enzyme catalysis, but it up-regulates gene expression levels^24^ and induces various medicinal effects. ARs are thus different from resveratrol in regard to the mechanism of SIRT activation.

Epidemiological studies have indicated that whole-grain intake is protective against cancer, cardiovascular disease, diabetes, and obesity^34^. Sirtuins’ enzyme activities are critical regulators of these diseases and also have primarily protective functions in the development of many age-related diseases^35^. Whole grains are rich in nutrients and phytochemicals with known health benefits^36^. ARs may have a central role in the whole grains for the function of these diseases. An open-label randomised trial study examined the effect of replacing refined wheat with whole-grain wheat for 12 wks on body weight and composition after a 2-wk run-in period of the consumption of refined wheat-containing food intake^37^, and the consumption of whole-grain products resulted in a greater reduction in the percentage fat mass without body weight changes.

On the other hand, ARs were also able to inhibit triglyceride accumulation in cultured 3T3-L1 cells, indicating that ARs may inhibit triglyceride synthesis *in vivo*^38^. These studies and our present findings indicate that the ARs may affect lipid metabolism in a sirtuin-dependent manner. ARs are present in an increasing number of organisms^39^, and their biological activity, their physiological role, and their participation in the regulation of metabolic processes are known only to a small extent. There is some evidence of non-linear associations between whole-grain intake and metabolic syndrome^40^. It is not clear that this association is related to ARs. The Mediterranean diet emphasises eating primarily plant-based foods, such as fruits and vegetables, whole grains, legumes and nuts. Much experimental evidence supports the suggestion that a Mediterranean diet may be beneficial with respect to reducing the incidence of metabolic syndrome^41^. The function of a Mediterranean diet may depend on the intake of ARs.

## Methods

### Extraction of the AR mixture from rye grain

Whole ground rye grains (3.6 kg) were extracted by continuous stirring for 24 hr at room temperature with 18 L of ethanol. A dried extract was dissolved in 50 ml of hexane. The soluble fraction was subjected to flash chromatography on a silica gel column (High-flash 5 L, 60 Å, 40 μm; Yamazen Science, Burlingame, CA) using step-gradient elution (v/v Hexane: EtOAc = 90:10, 9 min, 80:20, 15 min, 60:40, 15 min; flow rate: 70 ml/min, detection: 210 nm). The peaks from 32 min to 36 min were collected and evaporated to dryness. A collected sample was analysed by reversed-phase high-performance liquid chromatography (HPLC). The HPLC analysis of the ARs was achieved using an Inertsil ODS-3 C18, 5 μm, 4.6 × 250 mm column (GL Sciences, Tokyo) with 100% methanol.

### SIRT1 deacetylase assays by CycLex

The human SIRT1 deacetylase assays were performed using a CycLex SIRT1/Sir2 Deacetylase Fluorometric Assay Kit (CycLex, Columbia, MD, USA) according to the manufacturer’s instructions. In brief, the deacetylase reactions were carried out with recombinant SIRT1 and with various concentrations of NAD+ (12.5–200 μM) or fluoro-substrate peptide (0.5–20 μM) at room temperature. The fluorescence intensities were measured for 60 min at 1-min intervals using a microtiter plate fluorometer with excitation at 350 nm and emission at 450 nm.

### SIRT1 deacetylase assay with native p53 peptide monitored by HPLC

The SIRT1 reaction was carried out under the SIRT1 standard reaction conditions as described above with 1 μM native p53 peptide (Gly-Gln-Ser-Thr-Ser-Arg-His-Lys-(Ac-Lys)-Leu-Met-Phe-Lys-Thr-Glu-Gly; KareBay Biochem, Monmouth Junction, NJ) and 2.5 nM SIRT1 at room temperature for 30 min, yielding 10% conversion of the substrate to product, allowing for the accurate measurement of 10-fold activation. The HPLC separation of the acetylated p53 peptide substrate and deacetylated peptide product peaks was achieved using an Inertsil ODS-3 C18, 5 μm, 4.6 × 50 mm column (GL Sciences). The mobile phase A was 100% H_2_O with 0.1% trifluoroacetic acid, and mobile phase B was 100% CH3CN with 0.1% trifluoroacetic acid. The following gradient program was used: 0–35% over 15 min followed by a rapid ramping-up to 100% B to wash and then 100% A to re-equilibrate the column. The flow rate was kept constant at 1.0 ml/min, the column temperature was set at 25 °C, and the UV detection was set at 210 nm. The retention times of the deacetylated peptide and acetylated p53 peptide were 9.7 and 10.6 min, respectively.

### Histone deacetylation assays

Human monocyte THP-1 cells were grown in RPMI-1640 medium supplemented with 10% fetal bovine serum (FBS), 2 mM l-glutamine, and 50 μg/mL penicillin-streptomycin at 37 °C in a humidified chamber containing 95% air and 5% CO_2_. Semi-confluent cells were seeded at 1 × 10^7^ cells/well and then exposed to 100 nM trichostatin A (TSA), 10 μM olivetol, 1,3-dihydroxy-5-pentadecylbenzene (C15:0) (ReseaChem, Burgdorf, Switzerland), 1,3-dihydroxy-5-heptadecylbenzene (C17:0) (ReseaChem), and resveratrol (Cayman Chemicals, Ann Arbor, MI) for 24 hr. Acetylated histone was extracted with an EpiQuik Total Histone Extraction Kit (EpiGentek, Farmingdale, NY) according to the manufacturer’s instructions. Acetylated histone from the extracted total histone was measured with an EpiQuik Total Histone Acetylation Detection Fast Kit (EpiGentek) according to the manufacturer’s instructions.

### Quantitative real-time PCR assay

Human monocyte THP-1 cells were grown in RPMI-1640 medium supplemented with 10% FBS, 2 mM l-glutamine, and 50 μg/mL penicillin-streptomycin at 37 °C in a humidified chamber containing 95% air and 5% CO_2_. HepG2 cells (a human liver cancer cell line) were maintained in Eagle’s Minimum Essential Medium supplemented with 10% FBS, 2 mM l-glutamine, and 50 μg/mL penicillin-streptomycin at 37 °C in a humidified chamber containing 95% air and 5% CO_2_. To restrain glucose, THP-1 cells were cultured in glucose- free RPMI-1640 medium (Sigma-Aldrich, Tokyo) and HepG2 cells were cultured in glucose- free D-MEM medium (Wako Osaka). THP-1 and HepG2 cells were passage 4 times (20 population doublings) glucose restriction condition. In normal glucose (NG) condition, semi-confluent cells were seeded at 1 × 10^7^ cells/well and then exposed to 10 μM olivetol, 1,3-dihydroxy-5-pentadecylbenzene, 1,3-dihydroxy-5-heptadecylbenzene, and resveratrol for 6 hr. Quantitative reverse transcription-polymerase chain reaction (RT-PCR) was performed on a Thermal Cycler Dice Real-time PCR System (Takara, Shiga, Japan) according to the manufacturer’s instructions.

Total RNA was extracted from GR and NG culture condition cells with a guanidinium isothiocyanate-based RNA isolation kit (RNeasy Mini or RNAeasy Lipid Tissue kit; Qiagen, Valencia, CA) according to the manufacturer’s instructions. Two micrograms of RNA was converted into single-stranded DNA by a standard 50-μl RT reaction with a PrimeScript RT reagent kit (Takara). The cDNA generated from the reverse-transcription reactions was amplified by PCR with SYBR Premix Ex Taq™ II (Takara) in a total volume of 25 μl according to the manufacturer’s instructions. The primers used were as follows: β-Actin: 5′-TGCACCACACCTTCTACAATGA-3′/5′-CAGCCTGGATAGCAACGTACAT-3′; SIRT1: 5′-TAGAGCCTCACATGCAAGCTCTA-3′/5′-GCCAATCATAAGATGTTGCTGAAC-3′. The levels of each messenger RNA were expressed as the relative fold change versus the β-actin mRNA.

### Fly stocks, maintenance and adult longevity assay

A *w*^*1118*^ Drosophila line (stock no. 108479; identical to Iso31, isogenic *w*^*1118*^ stock in Bloomington Drosophila Stock Center [BDSC] #5905) was obtained from the Drosophila Genetic Resource Center (DGRC) and used as the wild-type experimental control. *Sir2*^*17*^ (stock no. 24857; genotype: *w*^*1118*^; *Sir2*^*17*^/*SM6a*) and *Sir2*^*KG00871*^ (stock no. 12966; genotype: *y*^*1*^ *w*^*67c23*^; *P{y* + ^*mDint2*^ *w*^*BR*.*E*.*BR*^ = *SUPor*-*P}Sir2*^*KG00871*^) were obtained from the BDSC. Trans heterozygous *Sir2*^*17*^/*Sir2*^*KG00871*^ is a fly in which *Sir2* is severely decreased^5^.

All of the flies were raised under a 12-hr light/dark cycle at 25 °C and 50% humidity on standard culture medium (standard food; SF) consisting of 10% (w/v) glucose, 7% (w/v) corn meal, 4% (w/v) yeast extract, and 0.55% (w/v) agar medium containing 0.3% (v/v) propionic acid and 0.35% (v/v) butyl p-hydroxybenzoate as antifungal agents as described^42^. The fly longevity assay was performed as described^42^.

## Additional Information

**How to cite this article**: Kayashima, Y. *et al*. Alkylresorcinols activate SIRT1 and delay ageing in *Drosophila melanogaster.* *Sci. Rep.* **7**, 43679; doi: 10.1038/srep43679 (2017).

**Publisher's note:** Springer Nature remains neutral with regard to jurisdictional claims in published maps and institutional affiliations.

## Figures and Tables

**Table 1:** Results of the lifespan trials of *D. melanogaster* adult males and females.

Trial	Genotype	Diet	Treatment	Females						
				N(0)	Median lifespan		Mean lifespan		Log-rank test	
					days	% change†	days	s.e.	χ ^2^	*p*-value
1	*w*^*1118*^	SF	Control	173	45		45.2	1.20		
			Resveratrol	173	49	**0**.**9**	48.7	1.44	9.53	0.0020
			ARs	173	57	**26**.**7**	55.0	1.29	36.7	<0.0001
2	SIR2 hypomorphism	SF	Control	102	65		59.8	1.74		
	*dSir2* [17]/KG00871		Resveratrol	38	60	*−0*.*83*	58.2	1.80	8.51	0.0035
			ARs	42	60	−0.83	52.7	3.71	0.25	0.62
									
1	*w*^*1118*^	SF	Control	250	47		44.6	0.92		
			Resveratrol	230	57.5	**22**.**3**	53.1	1.15	87.0	<0.0001
			ARs	250	57.5	**22**.**3**	54.4	0.90	92.1	<0.0001
2	SIR2 hypomorphism	SF	Control	154	57		52.9	1.22		
	*dSir2* [17]/KG00871		Resveratrol	40	53	**0**.**75**	50.5	1.83	10.1	0.0015
			ARs	113	57	0.0	54.3	1.42	1.09	0.30

^†^The percent change is relative to the control. Bold: increase in lifespan at *p* < 0.01. *Italics: decrease in lifespan* at *p* < 0.01. SF: standard food (see Methods).
